# Radiation Oncology: Future Vision for Quality Assurance and Data Management in Clinical Trials and Translational Science

**DOI:** 10.3389/fonc.2022.931294

**Published:** 2022-08-10

**Authors:** Linda Ding, Carla Bradford, I-Lin Kuo, Yankhua Fan, Kenneth Ulin, Abdulnasser Khalifeh, Suhong Yu, Fenghong Liu, Jonathan Saleeby, Harry Bushe, Koren Smith, Camelia Bianciu, Salvatore LaRosa, Fred Prior, Joel Saltz, Ashish Sharma, Mark Smyczynski, Maryann Bishop-Jodoin, Fran Laurie, Matthew Iandoli, Janaki Moni, M. Giulia Cicchetti, Thomas J. FitzGerald

**Affiliations:** ^1^ Department of Radiation Oncology, UMass Chan Medical School, Worcester, MA, United States; ^2^ Department of Biomedical Informatics, University of Arkansas, Little Rock, AR, United States; ^3^ Department of Biomedical Informatics, Stony Brook University, Stony Brook, NY, United States; ^4^ Department of Biomedical Informatics, Emory University, Atlanta, GA, United States

**Keywords:** radiation therapy (radiotherapy), clinical trials, cancer treatment, clinical trial imaging, clinical trial data, translational medicine, quality assurance, artificial intelligence

## Abstract

The future of radiation oncology is exceptionally strong as we are increasingly involved in nearly all oncology disease sites due to extraordinary advances in radiation oncology treatment management platforms and improvements in treatment execution. Due to our technology and consistent accuracy, compressed radiation oncology treatment strategies are becoming more commonplace secondary to our ability to successfully treat tumor targets with increased normal tissue avoidance. In many disease sites including the central nervous system, pulmonary parenchyma, liver, and other areas, our service is redefining the standards of care. Targeting of disease has improved due to advances in tumor imaging and application of integrated imaging datasets into sophisticated planning systems which can optimize volume driven plans created by talented personnel. Treatment times have significantly decreased due to volume driven arc therapy and positioning is secured by real time imaging and optical tracking. Normal tissue exclusion has permitted compressed treatment schedules making treatment more convenient for the patient. These changes require additional study to further optimize care. Because data exchange worldwide have evolved through digital platforms and prisms, images and radiation datasets worldwide can be shared/reviewed on a same day basis using established de-identification and anonymization methods. Data storage post-trial completion can co-exist with digital pathomic and radiomic information in a single database coupled with patient specific outcome information and serve to move our translational science forward with nimble query elements and artificial intelligence to ask better questions of the data we collect and collate. This will be important moving forward to validate our process improvements at an enterprise level and support our science. We have to be thorough and complete in our data acquisition processes, however if we remain disciplined in our data management plan, our field can grow further and become more successful generating new standards of care from validated datasets.

## Introduction

Radiation oncology has undergone fundamental change over the past several decades. The primary turning point in our discipline was the transition from anatomic to volumetric treatment planning pivoting away from previous two-dimensional planning methods. The transition required a seed change in how we identified disease and targeting of tumor volumes in juxtaposition to normal tissue which we could now evaluate in three and four dimensions. We became closer to our imaging colleagues but asked different questions of the images we review in collaboration. While imaging colleagues told us that a mass was present, radiation oncologists needed to know the size and peripheral location including the boundaries of involvement. We also needed to adapt our plans to our understanding of the natural history of disease and predictable routes of tumor spread including additional tissues at risk for disease. We need to balance our plans with our growing knowledge of normal tissue constraints and how systemic therapy modulates these constraints. We had to and continue to adapt our therapy to growing concerns of normal tissue pre-existing co-morbidity as society ages and need for radiation therapy in older populations becomes the sole option for treatment. Today there are innumerable medical coefficients to the management of each patient and the modern radiation oncologist must be fluent and knowledgeable in each detail of medical care.

Our technology has matured at a rapid rate, at times out pacing our ability to fully harness its strength and apply modern technology in a strategic manner to daily care. Historically, once the radiation oncologist left the simulator, most of the physician work was completed with less nuance and detail applied to treatment planning. We simply did not have tools to optimize our craft beyond fluoroscopy and two-dimensional treatment platforms. Today, the simulation appointment is designed to construct immobilization devices and provide four-dimensional imaging with fusion of diagnostic images as needed for target definition. The work of the radiation oncologist today only begins when the patient leaves the simulator. This has a profound impact on department workflow as the work of the physics planning team can only begin once the volumes to treat and constraints to follow are made available as part of the directive of care. If the patient requires rapid initiation of therapy and the contours are not completed in a timely manner, the physics planning team does not have the time to both optimize planning and perform quality assurance of the plan including the appropriate checks of the chart for patient care. Tools and strategy for daily image guidance are now an integrated component of patient care. Historically, we assumed it was self-evident that treatments were reproduced daily validated by a weekly mega electron volt (MeV) image. Today, directive for image guidance using volumetric and kilovoltage (kV) imaging coupled with optical tracking tools are standard of care. Magnetic resonance (MR) integrated tools monitor biological parameters of care and artificial intelligence tools are applied to predict outcome from radiomics and pathomics in multiple disease areas with protocols designed to both augment and titrate care based on evolving patient specific biomarkers.

The changes in both work scope and workflow in our discipline have been profound and continue to grow. Although the infrastructure of our skill set has roots with our first mentors, training in radiation oncology bears more limited resemblance to training programs of the past. The skill set required for the modern radiation oncologist is now broad, detailed, and requires comprehensive knowledge of medicine, surgery, radiology, and pathomics. Radiation oncology interacts on a near daily basis with every medical subspecialty, surgical subspecialty, radiology, pathology, and disease-based program within a cancer center. Radiation oncologists need to maintain the skill set of a surgeon for brachytherapy and simultaneously remain fluent in the pharmacokinetics of integrated systemic therapy for multi-disciplinary management. We must maintain expertise in imaging and applied pathology for biomarker assessment of developing treatment plans which may require dose titration or augmentation/dose painting. Radiopharmacy has the potential to mature into a powerful tool for both diagnosis and patient treatment. Modern care is challenging requiring constant communication between providers to ensure consistent messaging to patients and families.

We need to understand the strengths and limitations of our colleagues in oncology related disciplines and fill gaps in service and communication when appropriate. Every patient brings an opportunity for clinical research in tumor control and normal tissue outcome analysis, and we need to work harder at imbedding this activity into our daily work as part of our patient care management. We need to educate the next generation of providers and colleagues in primary care disciplines to recognize the fingerprints of therapy on normal tissue structure and function and optimize care as best as possible to prevent symptomatic normal tissue sequelae including the impact imposed on tissue by combined modality therapy. Medical education has begun to recognize the importance of oncology in medical practice establishing courses in oncology and oncology related patient care at several timepoints during each year of medical school. This will serve to provide common language between disciplines and promote improved understanding of oncology related matters.

Coupled with improvements in our discipline comes the responsibility of increasing our visibility in direct patient care and leadership in multi-disciplinary management. Radiation oncologists have historically and superficially been perceived through the prism of proceduralists and less involved with the longitudinal care of the cancer patient, often managed by colleagues in medical oncology. Today is a different day. Hepatocellular oncology, thoracic oncology, and central nervous system disease management invites multiple complex procedural based therapies including radiopharmacy directed care with radiation oncologists often assuming primary management for the coordination of care between medical and interventional radiology colleagues. Because of primary management of sub-total whole brain therapy, gastrointestinal presentations and pulmonary nodules of both primary and metastatic origin, radiation oncologists are following patients with equipoise previously associated with medical oncology in multiple disease areas. Interpretation of follow up therapy images in computed tomography (CT), positron emission tomography (PET), and magnetic resonance (MR) imaging require radiation oncology review to validate image interpretation relative to the radiation therapy treatment field. Often therapy leaves predictable changes on images which can be misinterpreted as disease. Accordingly, response assessment is often best accomplished in a multi-disciplinary setting including the fields of radiation therapy. This requires fingertip availability of radiation therapy treatment objects for review by colleagues outside of our discipline. Aside from improving patient care, this would also serve to educate our colleagues concerning process improvements in our discipline and educate trainees in other disciplines (1–5).

In this area, radiation oncologists are poised to assume more visible and influential leadership roles in disease-based disciplines. Our treatment has become more valuable to patient care due to improvements in tumor control and titration of sequelae of management. Because we are integrated with all liquid and solid disease systems with increasing patient care responsibility, we are maturing as thought leaders in oncology programs. This is both a strength and a potential weakness as we need to first mature and accept the responsibility as leaders and make certain our science and written manuscripts reflect the maturation of our discipline. Our clinical -translational science is improving and our basic science is drawing more attention and support at a national level. Our next objective is to move these functions to an enterprise level and establish integrated processes to move our science forward with validation. Process improvements in data acquisition and data management need to become part of our daily work.

## Responsibility of Clinical Trials and Translational Medicine

Radiation oncologists have participated in clinical trials sponsored by the National Clinical Trials Network’s (NCTN) former cooperative groups for more than 50 years. We have managed clinical trials as a primary discipline and participated in trials when the study required radiation therapy but not necessarily as the primary study question. Quality assurance in clinical trials initially centered in generating consistency in computational analytics. Prior to planning systems becoming more commercialized, field dose calculations were performed onsite with two-dimensional algorithms calculated at field isocenter or at depth. Phantoms were constructed by colleagues at the Radiological Physics Center (RPC, now IROC Houston) for protocols to generate consistency in computation and therapy execution across institutions. Fluoroscopic simulation images and images taken under megavoltage were submitted for quality assurance initially without diagnostic images to validate how the targets were chosen. Therapy fields were designed by anatomical considerations and were not necessarily driven by image guidance.

With the advent and development of three-dimensional planning systems, the paradigm shifted, and quality assurance moved beyond computational analytics generated through phantoms as a sole source identifying a plan as study compliant. Although consistency in computational algorithms remains important; harmonization through vendor technology facilitated consistent and reproducible approach to computations validating dose to volume. Equally relevant was the introduction of imaging directly into treatment planning and the skill set of the radiation oncologist pivoted towards a balance between imaging and computational analytical treatment planning algorithms. Diagnostic imaging colleagues often place focus on the presence or absence of a structure. Radiation oncologists needed to know the peripheral boundaries and three-dimensional shape of the target corresponding to normal tissue abutting the target. Radiation oncologists now had to think in terms of volumes and the relationship of target volumes to structure. Normal tissues likewise had volumetric measures and radiation oncologists now had to think and apply therapy with consideration to dose and volume both to disease and normal tissue. The dose volume histogram became an invaluable two-dimensional reconstruction of volumetric therapy and care plans could be compared through this prism. Therapy plans and full radiation oncology datasets could be shared, and protocols matured rapidly in the cooperative groups using volumetric language. These tools gave us voice and an opportunity for sharing information with providers and colleagues with common ground. More importantly, our discipline could speak in a more unified voice in a quantitative language germane to radiation oncology. Protocols matured with constraints to tumor targeting and normal tissue and provided us the opportunity to share information as colleagues in a digital format and intercompare outcome analysis with a common denominator. However, we understand our discipline does not function in isolation. Patient care and translational science require multiple disciplines to work in synergy and complement the strengths and weaknesses of other disciplines. We need to make certain colleagues in other direct and indirect patient care disciplines understand our technology and more importantly, the application of our technology to patient care and the meaning of radiation dose to volume. These processes affect all disease sites with opportunities for improvement in our science and patient care (1–21).

## The Need for Process Improvements in Our Clinical Translational Science

Clinical trials are mechanisms designed to improve patient care. Although clinical trials can be designed to ask direct questions in radiation therapy, often radiation oncologists participate applying radiation therapy in a uniform format in protocols evaluating chemotherapy and targeted therapy with radiation therapy. This is of equal importance to trials evaluating radiation therapy endpoints. Radiation therapy may not be the primary study question, however if not applied in a uniform manner, study questions may not be answered in the manner intended by the study design. The HeadSTART trial evaluated the role of the hypoxic cell sensitizer Tirapazamine in the management of patients with locally advanced head and neck carcinoma. There were multiple favorable phase 2 data supporting the use of the agent in management. The phase 3 trial was one of the first trials using volumetric planning in the management of patients with head and neck cancer. Because the trial was one of the first trials involving worldwide participation, the trial was managed with interventional review in near real time, but not pre-therapy real time as managed today with digital data exchange. Data including diagnostic imaging required to validate the choice of target volumes were submitted with radiation therapy treatment objects and was reviewed within the first three days of treatment. On review, radiation therapy quality had a direct impact on the results of the study and undermined the goal of the study. There were interesting caveats as patients of investigators who made adjustment on plans after the initiation of therapy had decreased survival compared to patients whose plans were approved on quality review *de novo*. These patients had survival similar to patients who were scored initially as study deviations but on retrospective review had volume deviations considered less clinically meaningful as the fields did not transgress gross tumor seen on imaging. This study demonstrated that the quality of radiation therapy mattered and despite our improved technology, we did not apply our technology in a uniform matter during an important study. Accordingly, the deviations overrode the primary study question and the utility of Tirapazamine as a hypoxic cell sensitizer could not be established. This demonstrated that we as a discipline had work to do to in supporting our colleagues in clinical trials, otherwise the benefits of our technology would remain less visible to oncology colleagues (10).

**Figure 1:**
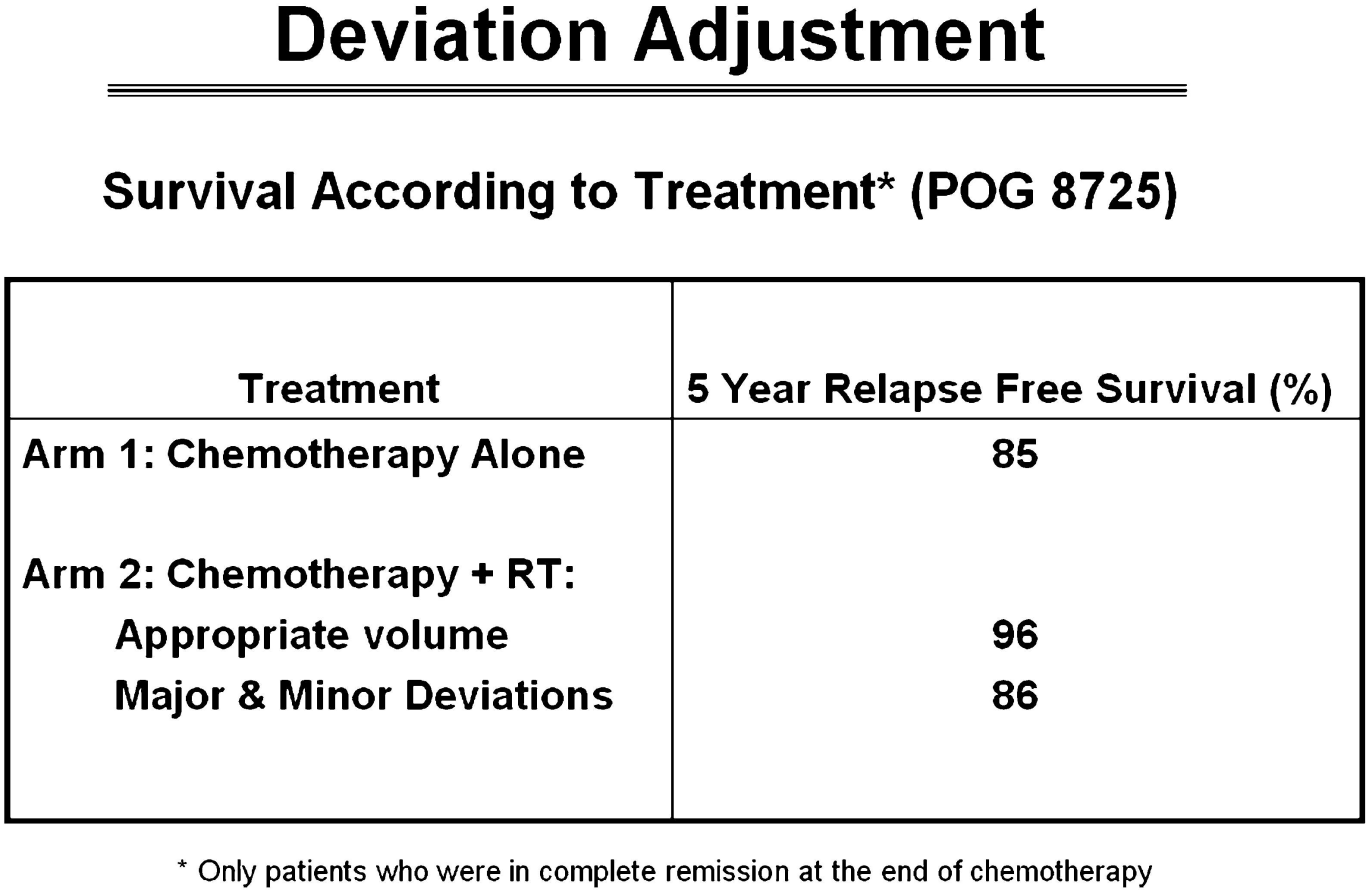
Non-protocol compliant radiation therapy had equal survival to patients treated with chemotherapy alone. Patients with protocol compliant radiation therapy had improved survival which was statistically significant (22).

A similar problem arose in Hodgkin lymphoma studies which revealed a survival benefit to patients treated with radiation therapy in a protocol compliant manner when the study did not demonstrate a benefit to radiation therapy as part of the primary evaluation due to the number of study deviations (**Figure 1**). Accordingly, we could not demonstrate our value in this disease. American College of Surgeons Oncology Group (now Alliance for Clinical Trials in Oncology) Z0011 breast cancer clinical trial was designed to assess the utility of surgical and radiation therapy titration of therapy to the axilla, however the lack of interventional review precluded uniform application of radiation therapy, thus did not answer the primary study point relative to radiation therapy. Today, axillary radiation therapy remains understudied and often misunderstood despite our multiple efforts to address these questions in clinical trials. This creates confusion among medical and surgical colleagues and became an opportunity lost to optimize care for these patients. Non-small cell lung carcinoma clinical trial formerly Radiation Therapy Oncology Group (RTOG) 0617 was designed as a two-tier randomization between low and high dose radiation therapy with systemic therapy for patients treated with definitive chemoradiotherapy. The trial did not show a benefit to higher dose radiation therapy and investigators moving forward assumed lower dose therapy was a standard of care. Less well known is the local control rate in the high dose arm was 12% less during the first three years of the study, possibly suggesting that tumor may/may not have been fully contoured as part of the gross tumor volume. Unfortunately, diagnostic imaging validating the contour of gross tumor was not collected, therefore the reason for the unanticipated result could not be evaluated. Therefore, an opportunity lost to ask an important question. If full diagnostic imaging datasets were reviewed as part of the study process, would trial outcome have been different? The trial has had and continues to have influence in the thoracic community as the results suggested that “less is better” when in fact there was no explanation for why the higher dose arm had worse local control in the initial management of the trial. We can only speculate that targeting of disease may have been incomplete due in part to concern of toxicity by investigators who may have unintentionally under contoured disease to spare parenchyma from toxicity. Today, in clinical trials evaluating the role of immunotherapy in lung cancer management assume that 60 Gray (Gy) to gross tumor is the standard of care. Although several other trials have suggested that higher dose may be optimal for local control, it remains challenging to convince others, including peers and insurance providers, that the trial may have had imperfections which influenced outcome. Therefore, despite our effort and good intentions, our trials have, at times, brought unintended downstream consequence to clinical management due to self-directed imperfections in data acquisition and data management drawing conclusions on studies that may or may not be accurate. Moving forward, we can only prove our point by collecting all data including outcome imaging and identify issues associated with local control and what can be done to both mitigate this point and not compromise normal tissue metrics. Constraints are influenced both by volume and intended dose and we need to study this in greater depth to become confident in our standards. These examples bring us to an understanding that as our discipline matures, we must accept greater responsibility for trial management and the narrative associated with the trial results if we are to be believed and recognized as thought leaders. We need to commit to normal tissue constraints in our trials and make effort to treat patients on trials abiding by normal tissue constraints to make accurate assessments of normal tissue tolerance. All too often, we let other disciplines control the narrative about radiation therapy. We need to mature as leaders with presence at interdisciplinary events where we can speak with respect but also speak from a leadership position. We need to not unintentionally contradict each other without understanding the context associated with the information. We must let the facts drive the narrative. Our presence at interdisciplinary national and international meetings will be increasingly important moving forward as we continue to treat a larger percentage of the oncology population. Our technology has matured, and we need to mature with our technology and represent the strengths of our discipline balanced with the inclusion of the strengths of colleagues. Our translational and basic science needs to continue to improve and this will improve our clinical trials. The National Cancer Institute recently established the core of a radiation oncology biology integrated network (ROBIN). This will promote the strengths of our science and provide visibility for our role in basic science and clinical trials. ROBIN will provide infrastructure to move our science into clinical trials. We need to integrate our science with current biomarkers, pathomics, and radiomics at an enterprise level as these vehicles become points of validation for our work and will serve to 1) identify patients of increased/decreased risk for recurrence, 2) interpret outcomes relative to science, and 3) improve clinical pathways for future patients and translational studies. A new initiative is being developed to house patient care data in a uniform format to develop programs in artificial intelligence in both our clinics and translational science laboratories. We understand that artificial intelligence will only be successful if built on strong datasets with complete information, otherwise we will be committed to continue to make the mistakes of the past limiting our credibility with colleagues from other disciplines (22–37).

## Next Steps

For our discipline to promote and apply our science in a meaningful manner to clinical trials, we will need to adapt our data management process. This will include all elements of information currently used to manage patients including outcome data with imaging to validate our performance and identify gaps for process improvements. The National Clinical Trials Network (NCTN) has tissue banks, clinical data, and outcome information associated with biomarkers housed in various platforms often within separate statistical centers. The Imaging and Radiation Oncology Core (IROC) supports the NCTN with imaging and radiation oncology data acquisition and management including real time review of objects to support the clinical objectives of study and the quality of treatment delivered during the clinical trial. These are important components to the infrastructure required to perform sound and modern translational science; however, each continues to function in relative isolation to each other with no natural pathway to generate interactions between the centers housing pathology, imaging, radiation therapy objects, and clinical outcome data. In addition, there are administrative layers which require approval to move data/information to investigators. Many within disease and discipline committees of the NCTN members point to the separation of data and redundant and duplicative effort required to retrieve data as a barrier to translational science involving secondary and unanticipated events associated with clinical trials. Accordingly, it remains challenging for investigators to bring all components necessary for research concerning secondary trial objectives not necessarily recognized at the time of trial development. Often, secondary questions, including review of quality assurance data, can only be optimally done at closure of the study and after the study has collected enough outcome information, including imaging, to assess questions concerning tumor control and toxicity (22, 34, 36, 37).

The Cancer Imaging Archive (TCIA) houses information on completed trials including clinical information, pathomics, imaging, and radiation therapy objects. TCIA has tools to permit research integrating all elements of data used for patient care and clinical trials. Investigators can apply established and novel tools for analysis including applications for the development of artificial intelligence to repurpose the data and ask questions not previously recognized during the conduct of the trial. The archive is rich and complete. The archives house all relevant information that can be rapidly retrieved for evaluation. Moving forward, TCIA will place enhanced emphasis on acquisition of radiation oncology objects including imaging used for target definition and outcome imaging for evaluation. With radiation therapy technology including theranostics rapidly moving forward, we need to accept the responsibility of making certain we collect and analyze all information to validate outcome and learn to appropriately apply our tools for patient care. If we do not collect all the relevant pathomic and radiomic information, we risk replicating our mistakes of the past and reach invalid conclusions as we have done far too often in previous trials as discussed. If we are to gain the most information from each trial, the trial must be conducted in a comprehensive manner with data transparency and sound acquisition processes to generate outcome analysis that can be trusted (38, 39).

Artificial intelligence models require validated and complete databases to develop strong algorithms which can serve as predictive indices for outcome. We will need artificial intelligence programs in all facets of our clinical, planning and research effort moving forward. The more complete the datasets used to build the models for artificial intelligence development, the more useful the models will be in clinical and academic practice. The challenge is to make this information available as quickly as possible to investigators. Although the data should naturally flow to an informatics platform that can be queried by investigators in near real time, the challenge remains that each group has the responsibility of data management and data protection, therefore data transfer to different programs requires approvals and integration of data flow processes between the centers of data acquisition for the information to be used and re-purposed in a meaningful manner. The databases have to be structured with a self-renewal process as images acquired more than a decade ago may or may not be relevant to a modern question as imaging platforms mature and diversify. Housing the information in the informatics library is important and the information must be curated to maintain relevance.

The TCIA infrastructure has been established at several institutions to serve as an institutional data management service as few platforms can store varied data and function at an enterprise level for review of information. This can serve as a platform to move data into the national archive. Radiation oncologists interact with all surgical, medical, imaging, pathology, and basic science colleagues daily. Our discipline needs to use our central position as caregivers as a strength and become leaders in data acquisition and management. It is only through this process can we provide opportunities for meaningful continuous self-improvement as our discipline is maturing a rapid pace. Having tools such as this housed within institutions gives investigators opportunities to review internal data and compare outcomes to information housed in the archive. Through this process we adjust and improve our skill and publish manuscripts with meaning and relevance. We can compare clinical drug x-ray interaction relative to normal tissue and functional metrics in similar clinical trials identified from the archive. Through these processes we can mature as a discipline, improve our metrics for normal tissue tolerance, and improve our definitions of risk categories and assign titrated or augmented therapies to patients within similar disease categories. The challenge is moving modern information with up-to-date imaging and pathomics to both IROC and TCIA platforms rapidly and in an enterprise manner. The biology data will include information obtained from modern pathomics including genomic sequencing and mutation analysis. The more we can streamline our processes, the more quickly we can build robust platforms to support our science. We need to build our departmental infrastructure to support data transfer in uniform formats to be repurposed for use in the next iteration of clinical science (38–50).

## Conclusion

Radiation oncology is no longer a lateral or secondary component in cancer management. We are important to current oncology management and are maturing as thought leaders in disease-based disciplines. Oncology patients will receive radiation oncology service today more than any other discipline in cancer management. We interact with patients from all disease sites and play a more prominent central role in the coordination of care for cancer patients We are a primary resource for follow up in many disease sites treated with advanced technology radiation therapy. The visibility we now possess comes with the responsibility of closing gaps in both our clinical care and translational science with equal attention to follow up care and management.

## Author Contributions

First author, LD. Senior author, TF. All authors contributed to the article and approved the submitted version.

## Conflict of Interest

Dr Ulin, Ms Smith, Ms Bishop-Jodoin, Ms Laurie, Mr Iandoli, Dr Moni, Dr Cicchetti, Dr FitzGerald report grants from the National Cancer Institute.

The remaining authors declare the absence of any commercial or financial relationships that could be construed as a potential conflict of interest.

## Publisher’s Note

All claims expressed in this article are solely those of the authors and do not necessarily represent those of their affiliated organizations, or those of the publisher, the editors and the reviewers. Any product that may be evaluated in this article, or claim that may be made by its manufacturer, is not guaranteed or endorsed by the publisher.
